# A Brief Review on Engaging and Interactive Learning for Children: Exploring the Potential of Metaverse-Based Oral Health Promotion

**DOI:** 10.1155/2024/6679356

**Published:** 2024-02-12

**Authors:** Vini Mehta, Ankita Mathur, Himanshu Chaurasia, Vishnu Teju Obulareddy, Cesare D'Amico, Luca Fiorillo

**Affiliations:** ^1^Department of Dental Research, Dr. D. Y. Patil Dental College and Hospital, Dr. D. Y. Patil Vidyapeeth, Pune 411018, Maharashtra, India; ^2^Virginia State Dental Association, Richmond, Virginia, USA; ^3^Department of Biomedical and Dental Sciences, Morphological and Functional Images, University of Messina, Messina 98100, Italy; ^4^Department of Dentistry, Faculty of Dental Sciences, University of Aldent, Tirana 1007, Albania; ^5^Multidisciplinary Department of Medical-Surgical and Odontostomatological Specialties, University of Campania “Luigi Vanvitelli”, Naples 80121, Italy

## Abstract

The importance of oral health for overall health makes it critical to establish proper oral hygiene practices in children early on. The traditional approaches to teaching children about dental health may not be successful since they may not be able to keep their interest. Metaverse technology offers a promising alternative, allowing for the design of engaging and immersive worlds that can effectively educate children about oral health. Despite the growing interest in the use of metaverse technology in healthcare, there is a lack of comprehensive reviews on its potential for oral health promotion in children. This review aims to fill this gap by providing an overview of the current state of metaverse-based oral health promotion for children, discussing its benefits and challenges, and highlighting its potential for improving children's oral health. By conducting this review, the authors hope to provide new information on the potential of metaverse-based oral health promotion for children and to contribute to the ongoing discussion on the use of metaverse technology in healthcare. This review may also provide valuable insights for dental organizations and practitioners interested in using metaverse technology to promote oral health and healthy living among children.

## 1. Background

Oral health is crucial for overall health, and promoting good oral hygiene habits from a young age is essential [1]. Traditional efforts to teach children about their dental health may not be effective because they may not be able to hold their attention or sustain their interest. Instead, children may find these methods boring and uninteresting. Metaverse is a 3D environment allowing users to interact with digital content and create avatars. It can be used for gaming, socializing, and education [2]. The metaverse is an online social space with many users that connects the real world to digital virtual worlds. Additionally, metaverse enables users to communicate with one another and their surroundings.

Different types of technology, such as augmented reality (AR) and virtual reality (VR), are used to achieve this goal. Users can enter the virtual universe through virtual reality devices thanks to the large-scale integration of AR, VR, and extended reality (XR) technologies. These situations can seem unrelated to our conventional teaching activities or may be restricted to the research laboratories. While online learning environments like Zoom, Teams, and Google meet have become common place, the development of virtual labs where actual participants are pushed to solve challenging scientific puzzles is also a reality [3, 4]. Scientists and tech firms are developing the metaverse to build a perceptual reality world that is most similar to the five senses of sight, touch, taste, hearing, and smell. Various fields like engineering, communication, arts, video games, entertainment, the financial sector, and even medicine and dentistry can employ this technology. Healthcare domains like medical education, diagnosis, treatment, clinical research, consultation, and holistic healthcare are all projected to be made easier by the metaverse platform, which consists of AR and VR glasses and a medical Internet of things (MIoT) system [5, 6].

The use of VR in dentistry is growing, with more research being conducted on creating and evaluating simulation models for use in the clinical settings and the shift from preclinical settings based on conventional models to clinics [7–9]. Although, there is scarcity of research in this advancing area of pediatric dentistry [5, 10, 11], it might be intriguing and instructive in regards to cases of trauma, dental anxiety, and oral cleanliness. With the help of metaverse, one can design engaging worlds for kids that are immersive and visually appealing. A few studies have applied metaverse on a children can, for instance, interact with virtual dentists, visit a virtual dental clinic, and engage in gamified activities to learn about oral health. These opportunities can enhance learning and promote involvement. For instance, a recent study of 2023 made a comparison between traditional physical therapy and physical therapy with metaverse in disabled children aged 8–15 years with cerebral palsy and it was reported to increase gross dexterity functions, heart-lung competence, and reduce the risk of COVID-19 transmission in children with disabilities [12–15]. Metaverse-based oral health promotion may be used in direct contact conversation as it simplifies oral hygiene education through engaging, interactive learning experiences. This mini-review aimed to provide an overview of the current state of metaverse-based oral health promotion for children with its benefits, drawbacks, and how it can be implemented.

## 2. Materials and Methods

An elaborative search was conducted in PubMed-MEDLINE, Scopus, Embase, Web of Science, and Google Scholar databases from inception till 30^th^ July, 2023. The articles were scrutinized based on the eligibility criteria of the review. The inclusion criteria for the study included peer-reviewed articles focusing on children aged 5–12 years, specifically addressing metaverse-based oral health promotion, assessing the effectiveness of metaverse-based interventions on oral health outcomes, and available in English language.

**Figure 1:**
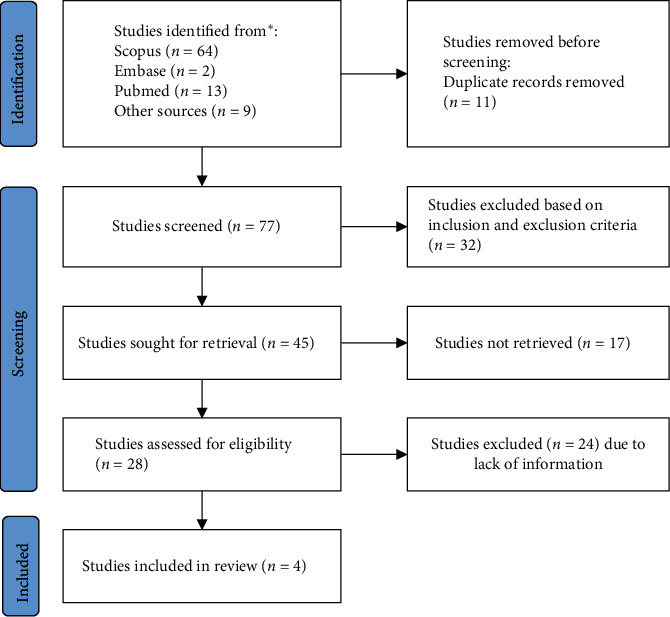
Identification of studies via databases and registers.

The search was performed using keywords such as “metaverse,” “oral health,” “dental health,” “children,” and “health promotion” using “OR” and “AND” Boolean operator. Two authors (V.M. and A.M.) did the literature search while a senior author (L. F.) verified it. The search results were screened for relevance by assessing the titles and abstracts, and the full texts of the selected articles were retrieved and critically reviewed. Key findings, themes, and recommendations were extracted and synthesized to provide a comprehensive overview of the topic. In addition to the database search, a manual search of relevant journals and conference proceedings was also conducted to identify additional relevant studies. The reference lists of the selected articles were also screened for additional relevant studies, and finally four articles were included in the study (Figure 1).

## 3. Findings

### 3.1. Metaverse and Oral Health

Metaverse technology is an emerging and promising area in the healthcare sector, with applications making an appearance [16–21]. It can effectively engage and educate children about oral health through interactive digital content, such as educational games and simulations [10]. This approach can be used by public or private health sectors to target groups and communities, providing accessible oral health education for school children, caregivers, and teachers [13, 14, 22, 23].

Motoric coordination development is crucial for children to learn about oral diseases and regulate their hygiene habits. Technology has made it easier to access educational videos, making them more enjoyable and memorable. Metaverse technology allows children to engage in a more realistic virtual world, learning oral hygiene in a simulation environment. Parents can also benefit from informational sessions about oral health in a virtual reality setting, increasing their knowledge and practice in oral hygiene [24]. Dental trauma is another common health problem that often affects children and adolescents. With the help of a trauma awareness simulation developed on the metaverse platform, parents and even children can learn about emergency treatments and facilities for dental trauma management [24].

Examples of metaverse-based oral health promotion for children include “Toothsavers” and “Happy Brushing.” “Toothsavers” is a fun and engaging game that educates children about good oral hygiene practices, while “Happy Brushing-Disney Magic Timer by Oral-B” is a virtual reality game that challenges players to brush a virtual set of teeth using appropriate procedures [10, 25, 26]. Summary of the articles is presented in Table 1.

### 3.2. Benefits of Metaverse-Based Oral Health Promotion for Children

**Figure 2:**
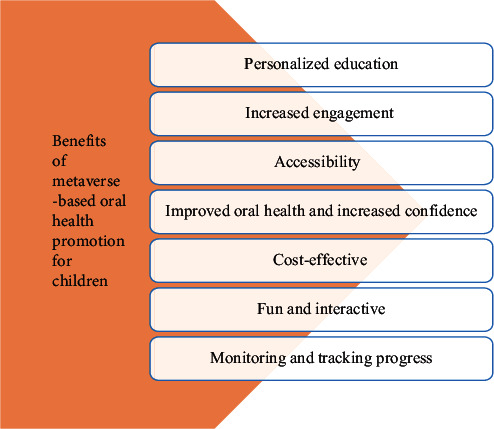
Benefits of metaverse based oral health promotion for children.

Metaverse-based oral health promotion has several benefits compared to the traditional methods. It is more participatory and appealing to children who may or may not have access to traditional resources, and more effective in teaching hard-to-reach children. Children who are afraid of the dentist may respond better to this approach and be more receptive to engaging with digital information about oral health [27]. Some key benefits include:

1. Personalized education: Metaverse-based learning offers personalized education by collecting data on children's interactions, tailoring content to their needs and preferences. This approach provides targeted exercises and feedback to help children improve in brushing areas, ensuring healthy oral hygiene habits are developed [28, 29].
2. Increased engagement: When information is delivered in a playful and engaging manner, children are more likely to pay attention and remember it [27, 30].
3. Accessibility: Children in remote areas or low-income communities who might not have access to traditional resources might nevertheless benefit from this strategy [28].
4. Improved oral health and increased confidence: Children who learn about good oral hygiene practices through metaverse-based oral health promotion may feel more confident in caring for their teeth and gums, lowering dental anxiety and enhancing general oral health [27].
5. Cost-effective: Creating digital content is frequently less expensive than using traditional methods, making it simpler for dental organizations and practitioners to reach a broader audience with minimal funds [28].
6. Fun and interactive: Metaverse-based oral health promotion is engaging and entertaining, which helps children to enjoy learning about dental health. This may lessen boredom and apathy toward the promotion and teaching of oral health. Children readily engage with the material and retain the knowledge if games and other interactive materials are included [27–29].
7. Monitoring and tracking progress: With the use of metaverse-based oral health promotion, parents, educators, and medical experts can keep track and provide them timely advice and guidance. This technique promotes regular oral health practices and aids in identifying areas [28]. All the benefits are highlighted in Figure 2.

### 3.3. Drawbacks of Metaverse-Based Oral Health Promotion for Children

**Figure 3:**
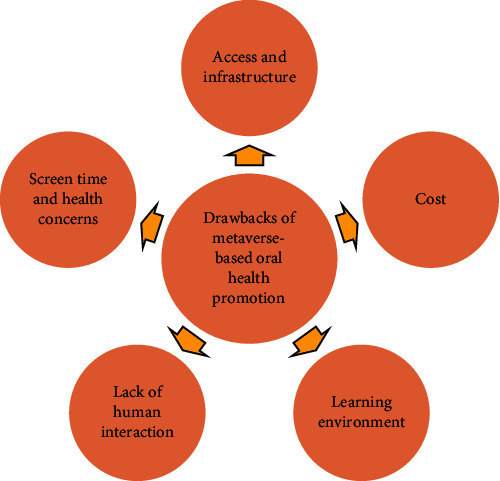
Drawbacks of metaverse based oral health promotion.

Although metaverse-based oral health promotion provides many advantages for children, there are also potential disadvantages to consider when comparing metaverse-based oral health promotion to traditional methods. Some of these disadvantages include:

1. Access and infrastructure: Limited infrastructure and access are some of the difficulties, which may hinder some children from participating in programs centered in the metaverse as not all may have these resources [29, 31].
2. Cost: The expense of executing these programs into action might be high, which restricts their uptake [29, 31].
3. Learning environment: Capacity of children to use these abilities in real-world circumstances may be impacted if the learning environment does not accurately reflect real-life events [29, 31].
4. Lack of human interaction: Metaverse-based learning may also be restrained by a lack of human interaction [29, 31].
5. Screen time and health concerns: Children spending excess time on screens may suffer from physical and mental health issues, and it may be challenging to transfer virtual experiences to real-world settings without additional support [29, 31]. Drawbacks of metaverse based oral health promotion are given in Figure 3.

### 3.4. Implementation of Metaverse-Based Oral Health Promotion for Children (Figure 4)

To effectively implement metaverse-based oral health promotion for children, several strategies must be considered. These include:

1. Collaboration between health sectors and metaverse developers: Developers of the metaverse should work with the public and private health sectors to produce tailored content and experiences that support the objectives of oral health education [32].
2. Integration into school curriculum: It can enhance accessibility and reach a larger number of children [33].
3. Enhancing access and infrastructure: It can be achieved through providing schools and community centers with necessary resources [32].
4. Balancing screen time: Limiting screen time is crucial, thus to address concerns about excessive screen time, it is important to maintaining a balance between metaverse-based learning and other activities [32].
5. Real-world application: Real-world situations should be incorporated to allow children to transfer knowledge gained in the metaverse to real-world situations [32].
6. Continuous monitoring and support: Regular check-ins, virtual consultations with dental specialists, and feedback mechanisms within the metaverse platform should all be part of continuous monitoring and support mechanisms [32].
7. Collaboration with dental professionals: Collaboration with dental specialists may make sure that experiences and content follow evidence-based guidelines and specifically address the oral health issues faced by children [32].
8. Parental involvement and education: Parental support, participation, and education are important to provide resources and learning materials to their children to grasp the advantages and potential disadvantages of this strategy [33].
9. Research and evaluation: The success of metaverse-based oral health promotion for children, including its influence on oral health outcomes, children's engagement and satisfaction, and the program's long-term sustainability, must be continually researched and evaluated [33].
10. Addressing equity and inclusion: Efforts should be taken to guarantee that oral health promotion programs built in the metaverse are inclusive and accessible for all kids, regardless of their socioeconomic status, geography, or physical capabilities [32]. For children who do not have access to the required technology or internet connectivity, alternate access methods must be made available. Some alternatives for children without access to technology or internet connectivity for oral health promotion include individual and group-based education strategies, population-wide mass media campaigns, and the use of mobile technologies [29].
11. Privacy and security: Given that metaverse-based programs are digital, it is crucial to give privacy and security measures top priority. Personal information about children should be secured, and suitable security measures should be in place to provide a secure online environment [32].
12. Educator training and support: To successfully use metaverse-based oral health promotion in their teaching practices, educators need proper training and support. Understanding the technology, the subject matter, and how to provide educational opportunities that are worthwhile for children are all part of this [33].
13. Feedback and iterative improvement: The metaverse-based oral health promotion program should regularly gather input from children, parents, educators, and dental professionals. By using an iterative approach, the program remains up-to-date, effective, and in line with the wants and preferences of its users [33].
14. Advocacy and policy support and: Supporting policies that encourage the incorporation of metaverse-based oral health promotion in educational and healthcare institutions can promote their wider acceptance and sustainability. Engaging stakeholders and policymakers can aid in establishing a supportive climate for the execution of these programs [33].

## 4. Discussion

The metaverse is a virtual environment that integrates various internet functions and services, including socializing, gaming, and business opportunities, within an immersive virtual reality universe. It has been a topic of debate, with some viewing it as the next logical step for humanity after the internet, while others believe it might have more negative effects [34]. One of the key benefits of the metaverse is its ability to connect people worldwide and eliminate physical distance, making it particularly beneficial for education [34]. Students can access immersive learning experiences and interact with peers and teachers in a 3D world [35]. The metaverse also facilitates greater learning speed and provides an inclusive environment for all learners [36] (36). It is important to note that the traditional methods such as videos or storybooks can still be effective in promoting oral health for children. Though, these traditional methods are limited in their ability to provide immersive experiences, lacking the interactivity and sense of presence that the metaverse provides. The metaverse offers a unique opportunity to engage children in a more immersive and interactive way, potentially leading to better outcomes in terms of oral health knowledge, behaviors, and attitudes. It also offers new opportunities for social interaction and collaboration. However, previous studies conducted on its application noticed a few drawbacks such as the availability of technology with adequate processing power and internet bandwidth for streaming videos is restricted for instructors and students. Glasses and AR/VR visors are required for an immersive experience. However, these are rather expensive. Extended usage may also result in adverse effects like weariness, dizziness, blurred vision, and irritation in the eyes [37]. Thus, it is crucial to carefully consider the potential benefits and drawbacks before fully embracing the metaverse.

## 5. Conclusion

Metaverse-based oral health promotion leverages new technology and digital platforms to engage children in novel and interesting ways. This keeps oral health education and promotion current with the newest trends and innovations. It also effectively engages and educates children about oral health through interactive digital content. This approach offers increased engagement and accessibility, making it a valuable tool for dental professionals and organizations. As technology advances, metaverse-based oral health promotion will become increasingly important in promoting oral health to children.

The metaverse-based oral health platform can empower children to take charge of their oral health and cultivate lifelong habits that will benefit them in the long run. It is anticipated to advance healthy lifestyles, well-being, good habits, while upholding health justice as core principles. While metaverse-based oral health promotion for children offers interesting possibilities, it is important to take into account any potential drawbacks and difficulties that can arise when putting it into practice.

By implementing the above strategies, metaverse-based oral health promotion may successfully educate and include children in oral hygiene practices, thereby improving oral health outcomes and overall well-being. Organizations and stakeholders may make the most of the advantages of metaverse-based oral health promotion while resolving any issues and ensuring the program's overall success. Thus, one can ensure that metaverse technology is used in a responsible and effective manner to promote children's dental health.

## Figures and Tables

**Figure 4:**
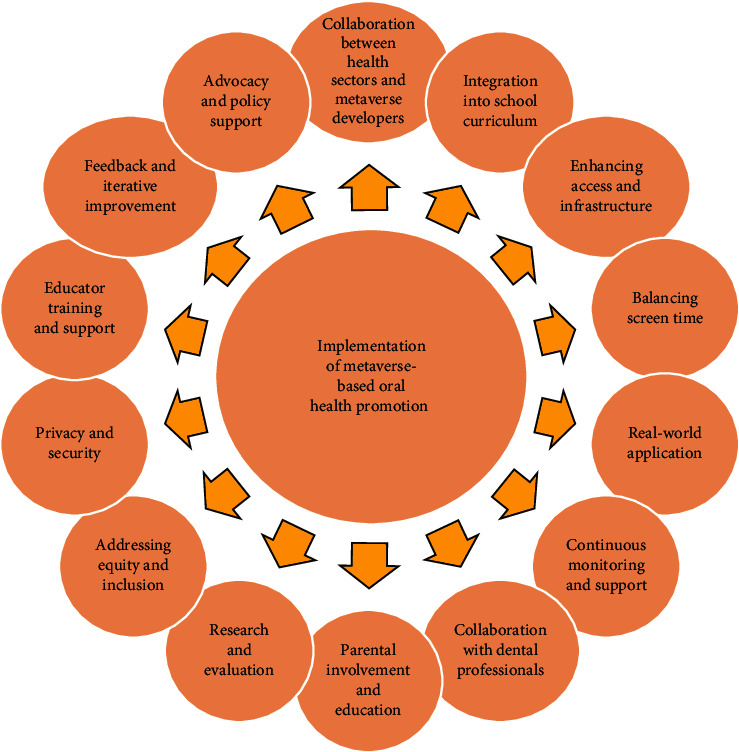
Implementation of metaverse based oral health promotion.

**Table 1:** Summary of the articles.

Author	Title	Type of the study	Main findings
Amantini et al.	Using augmented reality to motivate oral hygiene practice in children: protocol for the development of a serious game	Experimental study	This study describes the protocol for developing a serious game using augmented reality to motivate oral hygiene practice in children [10]

Felemban et al.	Effect of virtual reality distraction on pain and anxiety during infiltration anesthesia in pediatric patients: a randomized clinical trial	Randomized clinical trial	This study found that using virtual reality as a distraction during infiltration anesthesia in pediatric patients can reduce pain and anxiety [11]

Moon et al.	Effect of immersive virtual reality on pain in different dental procedures in children: A pilot study	Randomized clinical trial	This pilot study found that using immersive virtual reality can reduce pain in children undergoing different dental procedures [12]

Mehrotra and Markus	Emerging simulation technologies in global craniofacial surgical training	Review article	The technologies include 3D printed biomodels, virtual and augmented reality, use of google glass, hololens and haptic feedback, surgical boot camps, serious games, and escape games and how they can be implemented in low and middle income countries [22]

## Data Availability

Data for this research article are available on request to the corresponding author.
